# Prognostic value of amyloid/tau/neurodegeneration (ATN) classification based on diagnostic cerebrospinal fluid samples for Alzheimer’s disease

**DOI:** 10.1186/s13195-021-00817-4

**Published:** 2021-04-20

**Authors:** Koen Delmotte, Jolien Schaeverbeke, Koen Poesen, Rik Vandenberghe

**Affiliations:** 1 Department of Neurology, University Hospitals Leuven, Herestraat 49, B-3000 Leuven, Belgium; 2 Department of Neurology, Jessa Hospital, Hasselt, Belgium; 3 Laboratory for Cognitive Neurology, Leuven Brain Institute, KU Leuven, Leuven, Belgium; 4 Laboratory of Neuropathology, Leuven Brain Institute, KU Leuven, Leuven, Belgium; 5 Laboratory Medicine, University Hospitals Leuven, Leuven, Belgium; 6 Laboratory for Molecular Neurobiomarker Research, KU Leuven, Leuven, Belgium

**Keywords:** Alzheimer’s disease, ATN classification, Biomarkers

## Abstract

**Objective:**

The primary study objective of this retrospective academic memory clinic-based observational longitudinal study was to investigate the prognostic value of a cerebrospinal fluid (CSF)-based ATN classification for subsequent cognitive decline during the 3 years following lumbar puncture in a clinical, real-life setting. The secondary objective was to investigate the prognostic value of CSF biomarkers as continuous variables.

**Methods:**

Data from 228 patients (median age 67 (47–85) years), who presented at the Neurology Memory Clinic UZ/KU Leuven between September 2011 and December 2016, were included with a follow-up period of up to 36 months. Patients underwent a CSF AD biomarker test for amyloid-beta 1–42 (Aβ_42_)_,_ hyperphosphorylated tau (p_181_-tau) and total tau (t-tau) in the clinical work-up for diagnostic reasons. Patients were divided into ATN classes based on CSF biomarkers: Aβ_42_ for amyloid (A), p_181_-tau for tau (T), and t-tau as a measure for neurodegeneration (N). Based on retrospective data analysis, cognitive performance was evaluated by Mini Mental State Examination (MMSE) scores every 6 months over a period up to 36 months following the lumbar puncture. The statistical analysis was based on linear mixed-effects modeling (LME).

**Results:**

The distribution in the current clinical sample was as follows: A−/T−/N− 32.02%, A+/T−/N− 33.33%, A+/T+/N+ 17.11%, A+/T−/N+ 11.84%, A−/T−/N+ 4.39%, A−/T+/N+ 1.32% (3 cases), with no cases in the A−/T+/N− and A+/T+/N− class. Hence, the latter 3 classes were excluded from further analyses. The change of MMSE relative to A−/T−/N− over a 36-month period was significant in all four ATN classes: A+/T+/N+ = − 4.78 points on the MMSE; A−/T−/N+ = − 4.76; A+/T−/N+ = − 2.83; A+/T−/N− = − 1.96. The earliest significant difference was seen in the A+/T+/N+ class at 12 months after baseline. The effect of ATN class on future cognitive decline was confirmed for a different set of CSF thresholds. All individual baseline CSF biomarkers including the Aβ_42_/t-tau ratio showed a significant correlation with subsequent cognitive decline, with the highest correlation seen for Aβ_42_/t-tau.

**Conclusion:**

ATN classification based on CSF biomarkers has a statistically significant and clinically relevant prognostic value for the course of cognitive decline in a 3-year period in a clinical practice setting.

**Supplementary Information:**

The online version contains supplementary material available at 10.1186/s13195-021-00817-4.

## Introduction

Alzheimer’s disease (AD) is the most frequent cause of dementia, with a prevalence of approximately 0.2–0.9% in the age group of 60–64 years old, with an exponential increase to 10.7–13.1% in the age group of 80–84 years old [1].

Originally, for clinical diagnosis, the NINCDS-ADRDA diagnostic criteria [2] were used, with only a “probable” diagnosis based on the clinical presentation during life, as histopathological confirmation was essential for a “definitive” diagnosis. Newer criteria from the International Working Group [3, 4] and the NIA-AA (National Institute on Aging-Alzheimer’s Association) [5, 6] integrate Alzheimer’s specific in vivo biomarkers to support a clinical diagnosis, including cerebrospinal fluid (CSF) assessments.

In 2016, Jack et al. [7] proposed a new classification scheme based on biomarkers, for three dimensions: A/T/N. In this scheme, “A” stands for amyloid and is tested for by CSF amyloid-beta 1–42 (Aβ_42_) and/or amyloid positron emission tomography (amyloid-PET); “T” stands for tau and is tested for by CSF hyperphosphorylated tau (p_181_-tau) and/or a tau-PET; and “N” stands for neurodegeneration and is tested for by CSF total tau (t-tau), temporoparietal hypometabolism on [^18^F]fluodeoxyglucose PET ([^18^F]FDG-PET) or atrophy on magnetic resonance imaging (MRI). This ATN classification scheme is an unbiased descriptive system which can be applied to all patients, without being coupled to a specific diagnosis. The goal of this new classification system is to get insight in the evolution of the biomarkers in AD and eventually be able to make an early (even preclinical) biomarker-based diagnosis. Initially, it was intended for use in a research context, here we examine the prognostic value of this classification scheme in a clinical practice setting.

The temporal course of the abnormalities of biomarkers has been described before, during which an increase in amyloid load, characterized by reduced CSF Aβ_42_, precedes the spread of tau aggregates, neurodegeneration and cognitive decline [8–12].

In this retrospective academic memory clinic-based observational longitudinal study, we aimed to determine in a clinical context whether this new classification scheme can be used as a prognostic marker for the time course of cognitive decline. Secondarily, we wanted to investigate if there is a correlation between the continuous CSF biomarkers and the rate of deterioration of cognitive functioning.

## Materials and methods

### Patients

We included all cases who underwent a lumbar puncture for measuring CSF Alzheimer biomarkers for clinical diagnostic purposes, requested by the Neurology Memory Clinic UZ/KU Leuven in the period of September 2011 till December 2016 included. The end year for inclusion was chosen so as to allow for a total of 3 years of follow-up. The biomarkers tested were CSF Aβ_42_, p_181_-tau, and t-tau. AD CSF biomarker tests were performed for the evaluation of the presence of Alzheimer pathology in patients consulting the memory clinic where the etiological diagnosis of AD was considered based on clinical and/or neuropsychological assessment and MRI and a higher degree of diagnostic certainty was required. The most frequent reasons for seeking a higher degree of diagnostic certainty was age below 65; psychiatric, medical, or cerebrovascular comorbidity; or other confounding variables such as psychotropic medication, low reliability of cognitive assessment, e.g., due to language barrier, and atypical presentation such as primary progressive aphasia or corticobasal syndrome.

A list with the results of all CSF samples tested in UZ Leuven in this period was obtained, comprising 1125 samples, together with age at the time of the lumbar puncture and sex of the patients. To enhance homogeneity of the sample, we eliminated samples requested by external physicians or by other internal physicians who are not part of the Neurology Memory Clinic UZ/KU Leuven, so that a total of 410 samples remained. Medical records of these 410 patients were retrospectively analyzed for the Mini Mental State Examination (MMSE) scale (/30) [13] in a period of 6 months before the lumbar puncture (i.e., MMSE at time 0) and for every 6-month period up to a maximum of 3 years after the lumbar puncture (i.e., MMSE at time 6 for the period till 6 months after the lumbar puncture, time 12 for the period from 6 till 12 months, time 18 for the period from 12 till 18 months and so on until time 36, which corresponds to the period from 30 till 36 months after the lumbar puncture). Cases were only included if a baseline and at least two MMSE follow-up measurements were available. A total of 228 cases fulfilled this criterion, of which only three patients (1.32%) were classified as A−/T+/N+. None of the patients included can be considered cognitively normal as they all presented to the memory clinic for cognitive complaints and symptoms. The final clinical diagnosis was based on a comprehensive assessment consisting of clinical-neurological evaluation, neuropsychological assessment, MRI, and [^18^F]FDG-PET as well as CSF AD biomarker analysis. In the total group included in this report, the final clinical diagnosis was AD in 46.05% of cases. Other neurodegenerative disorders were diagnosed in 16–17% of patients (i.e., frontotemporal degeneration (behavioral variant and primary progressive aphasia) in 9.2%, Lewy Body disease in 4.4%, corticobasal syndrome in 1.8%, progressive supranuclear palsy in less than 1%), a non-neurodegenerative disorder in 28–29% of patients (psychiatric disorders in 7.0%, vascular dementia in 5.3%, internistic/toxic cause in 4.4%, other causes less frequent), and no clear diagnosis could be made in 8–9% of patients (Supplementary Table 8).

### Biomarkers

CSF biomarkers were measured with the INNOTEST assay (Fujirebio Europe, Ghent, Belgium). The cut-off values for classification were based on an in-house study performed in 38 asymptomatic individuals, using [^18^F]flutemetamol PET (amyloid-PET) as gold standard of amyloid-positivity [14]. In that study, the cut-off was determined that optimally discriminated amyloid-positive from amyloid-negative cases with a pre-specified specificity of 90% for each of three INNOTEST CSF biomarker variables separately as well as for the ratio of Aβ_42_ over total tau. The cut-off values were 798 pg/mL for Aβ_42_, 87 pg/mL for p_181_-tau, 465 pg/mL for t-tau, and 2.263 for Aβ_42/_t-tau [14]. These cut-off values correspond to a sensitivity for predicting amyloid PET positivity of 85.71% for Aβ_42_, 57.14% for t-tau, 42.96% for p_181_-tau, and 85.71% for Aβ_42_/t-tau. Based on these CSF biomarker cut-offs, patients were classified into binary ATN classes. These values also correspond to those used in clinical practice in UZ Leuven.

In the primary analysis, the cut-offs for p_181_-tau and t-tau were based on a comparison between amyloid-positive and amyloid-negative healthy controls. As a consequence, these cut-off values were relatively high. We examined the effect of lowering the p_181_-tau and t-tau cut-off on our results: we performed a secondary ROC analysis with a classification based on different p_181_-tau and t-tau thresholds derived from a clinical population, independent of the current study cohort. These lower cut-offs, derived from Youden’s index, were 58.9 pg/ml for p_181_-tau (specificity for discriminating a clinical cohort of AD patients from cognitively intact older adults = 75%, sensitivity = 82%) and 354 pg/ml for t-tau (specificity for discriminating a clinical cohort of AD patients from cognitively intact older adults = 70%, sensitivity = 89%). For the sake of comparison, the specificity and sensitivity of the original cut-offs for p_181_-tau (87 pg/mL) were 100% and 35.7% respectively, and for t-tau (465 pg/mL) 86.4% and 60%, respectively, when assessed in the independent cohort.

### Statistics

All statistical analyses were conducted with R statistical software, version 3.6.2 (2019-12-12) (The R Foundation for Statistical Computing) and R studio Version 1.2.5033. *P*-values were considered significant when meeting a two-tailed α threshold of 0.05. Correction for multiple comparisons was performed using Bonferroni, i.e., multiplying the *p*-value by the number of tests.

#### Descriptive statistics

A chi-squared test was used to determine whether sex was significantly different between ATN classes. Kruskal-Wallis analysis of variance was used to evaluate age differences between classes and differences in MMSE scores at baseline between ATN classes. In order to assess differences on MMSE score between ATN classes (five classes) at each time point (seven time points) separately, we additionally performed a Kruskal-Wallis test with post hoc Dunn test, which was Bonferroni-corrected for multiple comparisons.

#### Effect of ATN class on longitudinal MMSE course

To assess the effect of baseline MMSE score on subsequent MMSE scores, a linear regression analysis with baseline MMSE score as regressor and MMSE score on the consecutive time points (slope) as outcome measure was performed. We then calculated a linear mixed-effects model with random slope (LME-RS) using the “nlme” package in R software to investigate the evolution of MMSE over time in the different ATN classes. MMSE scores after baseline were used as dependent variable and the fixed effects predictor variables of interest were ATN class (with the A−/T−/N− profile as reference), time point of MMSE measurement, and the interaction term between ATN class and MMSE time point. This allowed to test whether cognitive decline differed in each ATN class relative to the reference population of A−/T−/N− and relative to the individual’s baseline MMSE. Case identity was used as a random intercept and a random slope was modeled for ATN class. Each model was corrected for age and sex. Using the R package “multcomp” we computed Tukey contrasts to compare lme-derived means between multiple groups. Missing values were coded as “na.omitted” in the lme model. For the group of 225 patients, 550 of the expected 1575 observations (34.92%) of data were missing. Therefore, we additionally calculated linear mixed effect models with random slope and imputation of missing values (LME-RS-IMV), for which missing values were estimated using multiple imputation with the mice package in R. CART was the imputation method used, which seeks to approximate the conditional distribution of a univariate outcome from multiple predictors (MMSE scores and ATN class). Five imputed datasets were calculated with the maximum number of iterations set at 50 and seed at 500. The missing values have been replaced with the imputed values in the first of the five datasets.

Using the alternative ATN classification approach, 217 patients were included in the analyses of which 526 of the expected 1519 observations (34.62%) of data were missing.

For visualization purposes, a MMSE slope was calculated using scores obtained at baseline and follow-up by means of latent growth curve analysis using the R package “lavaan” [15]. Missing MMSE scores were imputed using the CART imputation method as previously described. The slope was compared between ATN classes using Kruskal-Wallis and Dunn post hoc with Bonferroni correction.

As a secondary analysis, the cohort was divided based on syndrome diagnosis: mild cognitive impairment (MCI) or dementia (Supplementary Table 5). This classification was done using detailed investigation of clinical records by an experienced neurologist, taking into account anamnestic information and the Lawton instrumental activities of daily living (IADL) scale. LME-RS models were fitted within a group of 74 MCI patients (excluding 4 A−/T−/N+, 1 A−/T+/N+ due to low sample size of these ATN classes) and within 141 dementia patients (excluding 6 A−/T−/N+, 2 A-/T+/N+ due to low sample size), using imputation of missing data.

#### Effect of continuous CSF variables on longitudinal MMSE course

Besides the categorical ATN classification, we determined the effects of the continuous CSF biomarkers on the longitudinal MMSE course using linear mixed-effects modeling. The main analysis examined the effect of baseline Aβ_42_/t-tau on the MMSE course. Three additional analyses examined the effect of baseline Aβ_42_, baseline p_181_-tau, and baseline t-tau, respectively. For each individual CSF biomarker and for the ratio, MMSE score was used as outcome variable and the fixed effects predictor variables of interest were CSF biomarker and time point of MMSE measurement and an interaction term between CSF biomarker and time point. Case identity was used as a random intercept and the model was corrected for age and sex. Missing values were coded as “na.omitted” in the lme model. For the LME analysis with MMSE and CSF parameters, the MMSE slope correlation with each CSF parameter was visualized per ATN class using R studio Version 1.2.5033.

## Results

Data of a total of 228 patients were retrospectively analyzed, consisting of 119 (52.19%) male patients and 109 (47.81%) female patients. The baseline demographic characteristics across the different ATN classifications are shown in Table 1. At the moment of the lumbar puncture, patients were aged between 47 and 85 years old, with a median age of 67 years (Q1 = 60, Q3 = 72.25 years). There was no significant difference in age (*H*(5) = 5.70, *p* = 0.34) nor sex (*Chi squared*(5) = 3.62, *p* = 0.61) between ATN classes.

**Table 1:** Baseline characteristics of the different ATN classifications according to different cut-off values

ATN class	Number of patients (% of total)		Age range (median; Q1–Q3)		Sex male/female (% male)		Median MMSE at baseline
**A. Based on thresholds of 798 pg/mL for Aβ**_**42**_**, 87 pg/mL for p**_**181**_**-tau, and 465 pg/mL for t-tau**							
A−/T−/N−	73	(32.02%)	47–83	(67; 59–72)	41/32	(56.16%)	26
A+/T−/N−	76	(33.33%)	49–85	(66.5; 60–72)	39/37	(51.32%)	25.5
A+/T+/N+	39	(17.11%)	51–83	(69; 65–74.5)	20/19	(51.28%)	23
A+/T−/N+	27	(11.84%)	50–80	(68; 64–73)	11/16	(40.74%)	22
A−/T−/N+	10	(4.39%)	56–83	(64.5; 59.75–66)	7/3	(70%)	26
A−/T+/N+	3	(1.32%)	66–74	(71; 68.5–72.5)	1/2	(33.33%)	22
A−/T+/N−	0	(0%)	NA		NA		NA
A+/T+/N−	0	(0%)	NA		NA		NA
Total	228		47–85	(66; 60–72.25)	119/109	(52.19%)	25
**B. Based on thresholds of 798 pg/mL for Aβ**_**42**_**, 58.9 pg/mL for p**_**181**_**-tau, and 354 pg/mL for t-tau**							
A−/T−/N−	63	(27.63%)	47–83	(65; 58–72)	39/24	(61.90%)	25
A+/T−/N−	57	(25.00%)	49–85	(65; 60–72)	30/27	(52.63%)	26
A+/T+/N+	63	(27.63%)	51–83	(69; 64.5–74)	28/35	(44.44%)	26
A+/T−/N+	20	(8.77%)	50–79	(68.5; 60–72.5)	11/9	(55.00%)	23
A−/T−/N+	5	(2.19%)	66–76	(72; 67–72)	0/5	(0%)	27
A−/T+/N+	14	(6.14%)	56–83	(65.5; 62.5–67.75)	9/5	(64.29%)	26
A−/T+/N−	4	(1.75%)	61–74	(69.5; 65.5–72.5)	1/3	(25.00%)	26
A+/T+/N−	2	(0.88%)	75–78	(76.5;75.75–77.25)	1/1	(50.00%)	26
Total	228		47–85	(66; 60–72.25)	119/109	(52.19%)	25

*ATN* amyloid/tau/neurodegeneration, *MMSE* Mini Mental State Examination, *NA* not applicable

**Fig. 1:**
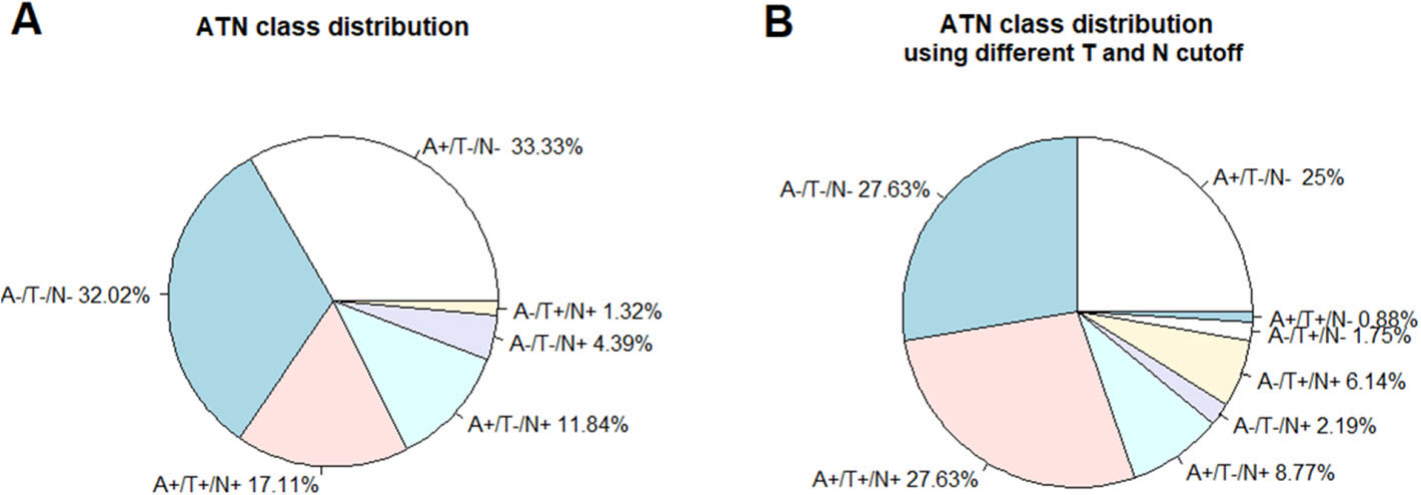
The pie chart illustrates the distribution of each ATN class in a memory-clinic derived sample of patients. **a** The cut-off values used to define the ATN classes were 798 pg/mL for Aβ_42_, 87 pg/mL for p_181_-tau, and 465 pg/mL for t-tau. **b** The cut-off value is the same for Aβ_42_, but lowered for tau: 58.9 pg/mL for p_181_-tau, 354 pg/mL for t-tau

The amyloid-only positive class (A+/T−/N−) and the triple-negative class (A−/T−/N−) were by far the largest classes, consisting of respectively 76 and 73 patients, which corresponds to 33.33% and 32.02% of patients in our study population (Fig. 1a). The triple-positive class (A+/T+/N+) consisted of 39 patients (17.11%). Twenty-seven patients (11.84%) were classified as A+/T−/N+, 10 patients (4.39%) as A−/T−/N+, and only 3 patients (1.32%) as A−/T+/N+ (Fig. 1a). Two classes were not represented in our study, namely A−/T+/N− and A+/T+/N. The underpowered A−/T+/N+ class as well as the two empty classes (A−/T+/N− and A+/T+/N−) were omitted in the further statistical analyses, resulting in a total sample of 225 patients.

With the alternative ATN classification approach using lower p_181_-tau and t-tau thresholds, the A+/T+/N+ increased in number and became approximately as frequent as A−/T−/N− and A+/T−/N− (Fig. 1b).

The baseline MMSE at time 0 was significantly different between ATN classes (Kruskal-Wallis chi-squared *H*(4) = 23.36, *p* < 0.001). When comparing to the triple-negative class, the median baseline MMSE was significantly lower for the triple positive class (*p* = 0.004) and A+/T−/N+ (*p* = 0.001), as shown in Table 1.

A linear regression analysis with baseline MMSE score as regressor and MMSE slope on the consecutive time points as outcome measure was statistically significant within the triple-negative class (*p* = 0.002) and in the A+/T−/N+ group (*p* = 0.006).

### Effect of ATN class on longitudinal course of MMSE

**Fig. 2:**
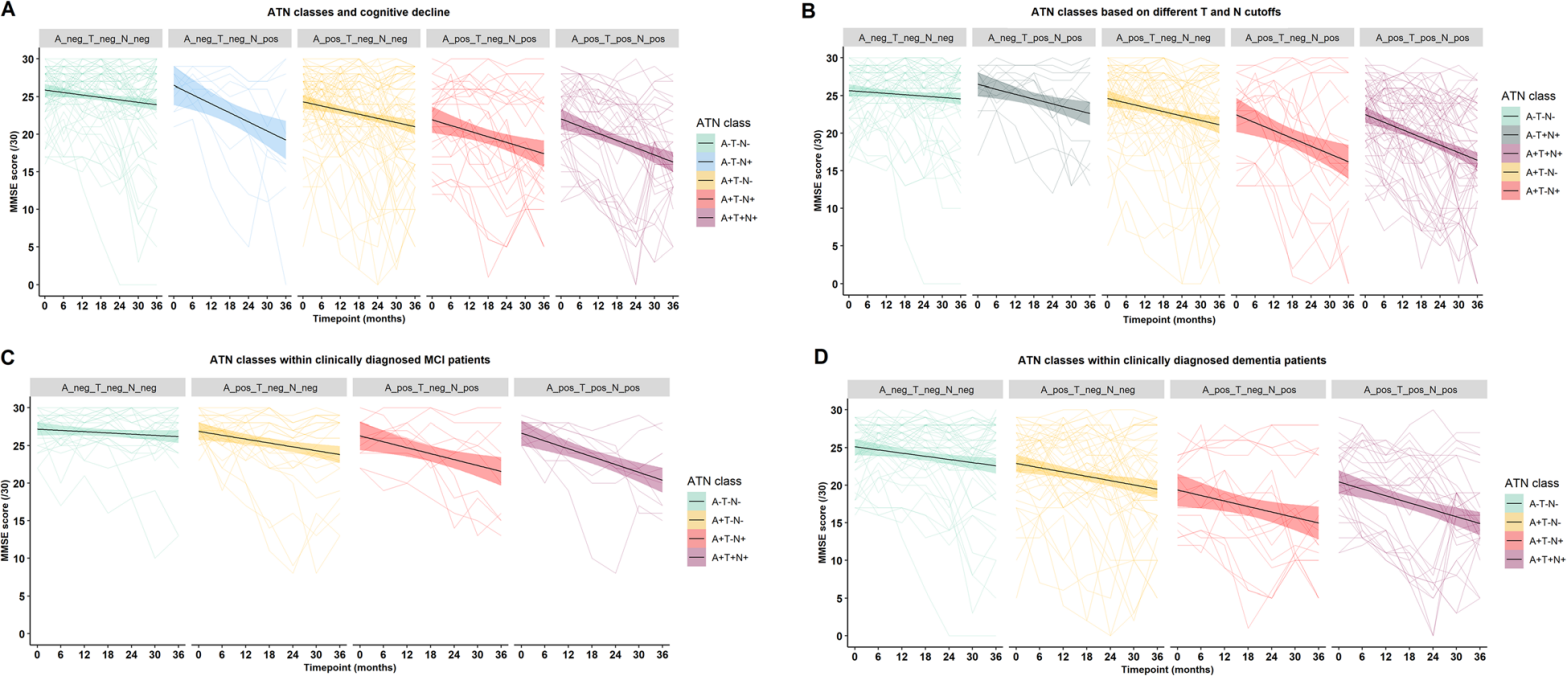
Evolution of MMSE according to ATN classes over a 36-month follow-up period, averaged per ATN class. **a** The cut-off values used to define the ATN classes were 798 pg/mL for Aβ_42_, 87 pg/mL for p_181_-tau, and 465 pg/mL for t-tau. **b** The cut-off value is the same for Aβ_42_, but lowered for tau: 58.9 pg/mL for p_181_-tau, 354 pg/mL for t-tau. Note that A−/T−/N+ is no longer included in the second panel, but now A−/T+/N+ is included, hence the different color used in the second plot. **c** Evolution of MMSE in the MCI subgroup. **d** Evolution of MMSE in the dementia subgroup. Standard errors are represented in colored zones for each ATN class. MMSE, Mini Mental State Examination

**Fig. 3:**
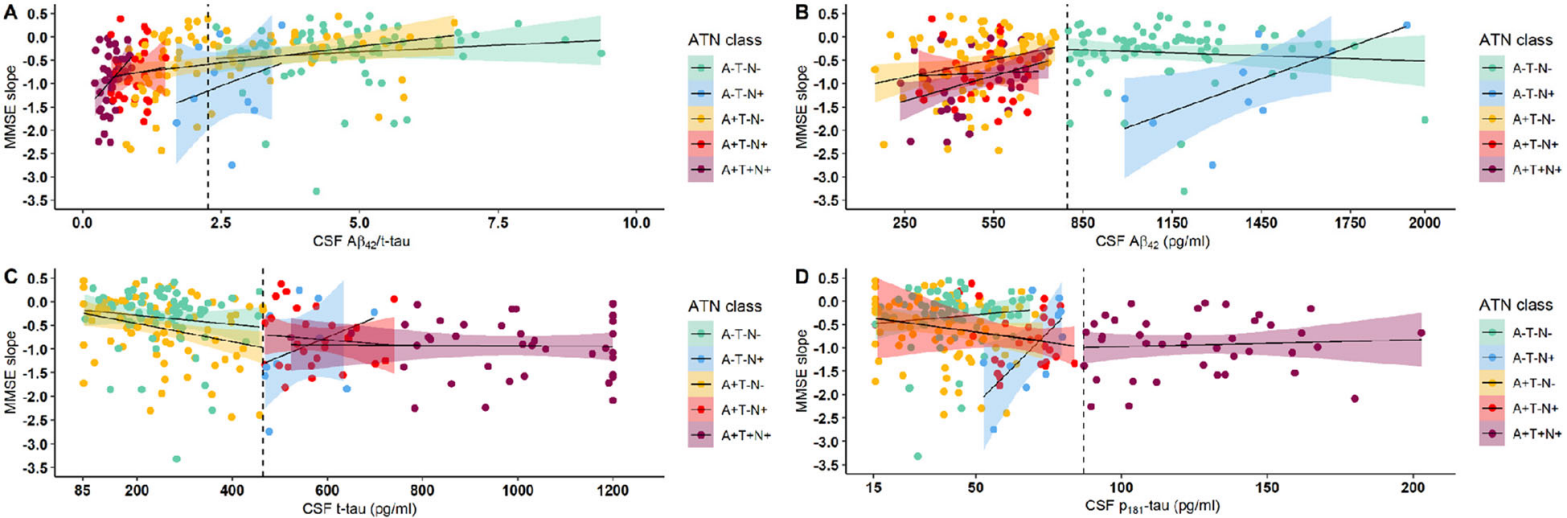
Interaction of individual continuous CSF biomarkers on the slope of MMSE within ATN classes. The more negative the value of the slope, the steeper the cognitive decline. **a** The effect of CSF ratio Aβ_42_/t-tau. **b** The effect of Aβ_42._ **c** The effect of CSF t-tau. **d** The effect of p_181_-tau. The dashed line corresponds to the CSF cut-off for each particular biomarker. MMSE, Mini Mental State Examination; CSF, cerebrospinal fluid; Aβ_42_, amyloid-beta 1–42; t-tau, total tau; p_181_-tau, hyperphosphorylated tau at threonine 181

Table 2 shows the evolution per time point for the different ATN classes compared to the reference class (A−/T−/N−). The values are derived from the linear mixed-effects model with random slope (LME-RS). Results from the linear mixed-effects model with random slope and imputation of missing values showed very similar results (Supplementary Table 1). Results are visualized in Figs. 2 and 3.

**Table 2:** Difference in MMSE at different time points relative to the reference class (A−/T−/N−) and to the baseline MMSE using linear mixed-effects model with random slope

**ATN class (*n*)**	**Time point**	**Difference in MMSE relative to baseline value**	**95% CI**	***t***	***P***
A−/T−/N− (*n* = 73)	Time 6	0.53	−0.59, 1.66	0.91	0.3629
	Time 12	0.03	−1.07, 1.13	0.05	0.9640
	Time 18	−0.05	−1.22, 1.12	− 0.08	0.9343
	Time 24	−0.86	−1.99, 0.27	− 1.47	0.1417
	Time 30	− 1.91	−3.22, −0.60	−2.81	**0.0050**
	Time 36	−1.29	− 2.60, 0.02	− 1.90	0.0580
**ATN class (*n*)**	**Time point**	**Difference in MMSE relative to value predicted based on A−/T−/N− at that time point and baseline MMSE**	**95% CI**	***t***	***P***
A+/T−/N− (*n* = 76)	Time 6	−0.76	−2.37, 0.85	− 0.90	0.3662
	Time 12	−1.04	− 2.55, 0.47	− 1.33	0.1851
	Time 18	−1.51	−3.06, 0.04	−1.88	0.0611
	Time 24	−2.24	−3.80, −0.68	− 2.77	**0.0058**
	Time 30	−1.87	−3.57, −0.17	−2.12	**0.0347**
	Time 36	−1.96	−3.73, −0.18	−2.13	**0.0339**
A+/T+/N+ (*n* = 39)	Time 6	−1.35	−3.18, 0.49	−1.41	0.1583
	Time 12	−2.16	−3.89, −0.42	− 2.39	**0.0169**
	Time 18	−3.97	−5.77, −2.16	−4.24	**0.0000**
	Time 24	−3.71	−5.58, −1.83	− 3.81	**0.0001**
	Time 30	−4.11	−6.18, −2.05	−3.84	**0.0001**
	Time 36	−4.78	−6.97, −2.58	− 4.20	**0.0000**
A+/T−/N+ (*n* = 27)	Time 6	−0.39	−2.73, 1.96	−0.32	0.7500
	Time 12	−0.28	−2.25, 1.68	−0.28	0.7813
	Time 18	−1.73	−3.78, 0.32	−1.63	0.1034
	Time 24	−1.62	−3.69, 0.46	−1.50	0.1334
	Time 30	−1.18	−3.41, 1.05	−1.02	0.3090
	Time 36	−2.83	−5.04, −0.62	−2.47	**0.0136**
A−/T−/N+ (*n* = 10)	Time 6	−0.66	−3.88, 2.56	−0.40	0.6927
	Time 12	−1.76	−4.70, 1.19	−1.15	0.2504
	Time 18	−2.08	−5.31, 1.15	−1.24	0.2148
	Time 24	−5.25	−9.36, −1.14	−2.46	**0.0140**
	Time 30	−8.79	−15.39, −2.20	−2.57	**0.0103**
	Time 36	−4.76	−9.65, 0.12	−1.88	0.0605

*MMSE* Mini Mental State Examination, *CI* confidence interval. The model is corrected for age and sex. Time 6 corresponds to the period 0–0.5 year; time 12 corresponds to the period 0.5–1 year; time 18 corresponds to the period 1–1.5 year; time 24 corresponds to the period 1.5–2 year; time 30 corresponds to the period 2–2.5 year; time 36 corresponds to the period 2.5–3 year. Significant *p*-values are represented in bold

The MMSE in the triple-negative class (A−/T−/N−) dropped by just over one point at time 36. The results of all other classes are to be interpreted as the extra difference in MMSE, compared to what one would predict based on the time course of the reference group (A−/T−/N−) and the individual’s initial MMSE. For instance, for the triple-positive class (A+/T+/N+), the MMSE significantly decreased relative to the triple-negative class (A−/T−/N−) starting already at 12 months. At 36 months, a decrease of 4 to 5 points relative to the reference class was estimated, corresponding to an absolute drop in MMSE of 5 to 6 points. The MMSE slope differed significantly between ATN classes over a 36-month period (*chi squared* = 31.65, *p* < 0.0001). Post hoc comparisons showed that the MMSE slope was significantly less steep in the triple-negative class compared to A+/T−/N+ (*p*_corr_ = 0.034). No other group comparison reached significance after correction for multiple comparisons. For further details, we refer to Table 2 and Fig. 2a.

With the alternative ATN classification scheme based on lower p_181_-tau and t-tau cut-offs, the results were essentially the same: the A+/T+/N+ group showed a significant decline compared to the A−/T−/N− group from month 12 onwards (extra decline in MMSE at 12 months = − 1.86 (CI −3.43, − 0.30)), the A+/T−/N+ group from month 18 onwards (extra decline in MMSE at 18 months = − 3.20 (CI −5.47, − 0.93)), and the A+/T−/N− group from month 24 (extra decline in MMSE at 24 months = − 2.03 (CI − 3.76, − 0.30)). This confirms the predictive value of the CSF-based ATN classification for rate of cognitive decline independently of the exact way the thresholds are calculated (within reasonable limits) (Fig. 2b) (Supplementary Tables 2 and 3).

When cognitive decline was fitted within the MCI subgroup: both the A+/T−/N− group as well as the A+/T+/N+ group started to decline at 18 months compared to the A−/T−/N− group. This decline in the A+/T+/N+ group was almost double in magnitude compared to the A+/T−/N− group (extra decline in MMSE at 18 months = − 2.19 (CI −3.97, − 0.40) in A+/T+/N+, and − 4.20 (CI −6.63, − 1.77) in A+/T−/N−). The A+/T−/N+ group started to decline from month 36 onwards (extra decline in MMSE at 36 months = − 3.30 (CI − 5.73, − 0.87)) (Supplementary Table 6). Numerically, from 18 months onwards, the decline in the A+/T+/N+ group was stronger than in any of the other groups at each of the subsequent timepoints. There was a difference of more than 3 MMSE points at 36 months between A+/T+/N+ versus A+/T−/N−.

Within the group clinically diagnosed with dementia, the A+/T+/N+ group started to decline from month 12 onwards compared to the A−/T−/N− group (extra decline in MMSE at 12 months = − 2.59 (CI −4.68, − 0.51)). At 18 months, the A+/T−/N+ group declined (extra decline in MMSE at 18 months = − 3.21 (CI −5.71, − 0.72)), while the A+/T−/N− group approached a nearly significant decline as well (Supplementary Table 7). Numerically, the decline in the A+/T+/N+ group was stronger than in any of the other groups at each of the timepoints, with a 2.5 MMSE points difference at the final 36 months timepoint between A+/T+/N+ versus A+/T−/N−.

### Effect of continuous CSF variables on longitudinal MMSE course

For the secondary analysis, a significant effect of continuous CSF baseline values on the time course of MMSE scores for Aβ_42_/t-tau ratio and also for each of the three CSF biomarkers separately was shown. This effect appeared at month 18 and remained significant until month 36 (*p* < 0.001 at time 36 for the different biomarkers), as is shown in Table 3. The Aβ_42_/t-tau ratio showed the strongest effect (effect size 0.61 (CI 0.25, 0.98; *p* = 0.0012). Results for the other biomarkers are as follows: effect size index of 0.0029 (CI 0.0009, 0.0049; *p* = 0.0048) for Aβ_42_, − 0.0037 (CI −0.0060, − 0.0013; *p* = 0.0031) for t-tau, and − 0.024 (CI −0.045, − 0.0040; *p* = 0.020) for p_181_-tau. Results from the linear mixed-effects model with random slope and imputation of missing values showed very similar results (Supplementary Table 4). For visualization purposes, the MMSE slope is plotted against the individual continuous CSF variables in Fig. 3. Independent of the ATN classification, a higher CSF Aβ_42_/t-tau ratio (*p* < 0.0001), higher CSF Aβ_42_ (*p* = 0.0013), lower p_181_-tau (*p* = 0.0005) and lower t-tau (*p* = 0.0001) were associated with a slower decline in MMSE (*p*-values represent values at 3 years, although significant differences are already noted starting from time 18 as shown in Table 3). Within each ATN class, globally the same effects are observed, with exception of a strong positive correlation between reduced cognitive decline (i.e., a more positive slope) with increased p_181_-tau levels (Pearson *rho* = 0.66, *p* = 0.038) in the A−/T−/N+ group (Fig. 3d). The apparent inverse correlation between MMSE slope and t-tau did not reach significance (Pearson *rho* = 0.35, *p* = 0.32).

**Table 3:** Interaction of individual CSF biomarkers on evolution of MMSE using linear mixed-effects model

CSF biomarker	Time point	Interaction	95% CI	***t***	***P***
Aβ_42_/t-tau	Time 6	0.1995	− 0.1375, 0.5365	1.1532	0.2492
	Time 12	0.3142	0.0002, 0.6282	1.9489	0.0517
	Time 18	0.7158	0.4114, 1.0201	4.5804	**0.0000**
	Time 24	0.7278	0.3980, 1.0576	4.2976	**0.0000**
	Time 30	0.6932	0.3525, 1.0339	3.9628	**0.0001**
	Time 36	0.8985	0.5423, 1.2548	4.9123	**0.0000**
Aβ_42_	Time 6	0.0011	−0.0006, 0.0028	1.2622	0.2073
	Time 12	0.0013	− 0.0004, 0.0030	1.5417	0.1236
	Time 18	0.0028	0.0011, 0.0046	3.2476	**0.0012**
	Time 24	0.0030	0.0012, 0.0049	3.1770	**0.0015**
	Time 30	0.0030	0.0009, 0.0052	2.7235	**0.0066**
	Time 36	0.0035	0.0014, 0.0056	3.2335	**0.0013**
p_181_-tau	Time 6	−0.0084	−0.0252, 0.0085	−0.9701	0.3323
	Time 12	−0.0133	−0.0294, 0.0029	−1.5972	0.1106
	Time 18	−0.0267	−0.0438, − 0.0096	−3.0428	**0.0024**
	Time 24	−0.0225	−0.0398, − 0.0052	−2.5298	**0.0116**
	Time 30	−0.0220	−0.0405, − 0.0036	−2.3239	**0.0204**
	Time 36	−0.0376	−0.0585, − 0.0168	− 3.5146	**0.0005**
t-tau	Time 6	−0.0009	− 0.0030, 0.0011	− 0.8744	0.3821
	Time 12	−0.0018	−0.0037, 0.0002	−1.7530	0.0800
	Time 18	−0.0042	−0.0062, − 0.0022	−4.1350	**0.0000**
	Time 24	−0.0035	−0.0056, − 0.0014	−3.2733	**0.0011**
	Time 30	−0.0036	−0.0058, − 0.0014	−3.1591	**0.0016**
	Time 36	−0.0050	−0.0075, − 0.0026	−4.0395	**0.0001**

*CSF* cerebrospinal fluid, *CI* confidence interval. Model is corrected for age and sex. Time 6 corresponds to the period 0–0.5 year; time 12 corresponds to the period 0.5–1 year; time 18 corresponds to the period 1–1.5 year; time 24 corresponds to the period 1.5-2 year; time 30 corresponds to the period 2–2.5 year; time 36 corresponds to the period 2.5–3 year. Significant *p*-values are represented in bold

## Discussion

In this retrospectively analyzed dataset of 225 patients who underwent a lumbar puncture for diagnostic purpose to measure CSF AD biomarkers in a clinical practice context, the triple-negative class (A−/T−/N−), the amyloid-only positive class (A+/T−/N−), and the triple-positive class (A+/T+/N+) were the dominant classes (Fig. 1). The conclusion that CSF-based ATN classification has predictive value for cognitive decline in a real-life clinical setting remained valid, regardless of varying the p_181_-tau and t-tau cut-offs within reasonable limits.

Over the period of 3 years, MMSE decreased significantly more in all represented classes relative to the reference class A−/T−/N−. In the triple-positive class (A+/T+/N+), MMSE decreased by almost 5 points more than the triple-negative class (A−/T−/N−) after 3 years and started to differ significantly from the reference class at 12 months. At 24 months, MMSE also started to decrease significantly in the amyloid-only positive (A+/T−/N−) and neurodegeneration-only positive (A−/T−/N+) classes, with a relative decrease at 36 months of respectively 2 and 5 points compared to the reference group. At 36 months, the A+/T−/N+ class also had a significant decrease in MMSE of almost 3 points compared to the triple-negative class. However, these values may not be representative for the general population seen at the memory clinic as a CSF diagnostic test was only done for specific indications. This may theoretically have introduced a selection bias for cases more likely to have a more rapid progression.

When taken into account the syndrome diagnosis, the cognitive decline is earlier and more pronounced in the dementia group versus the MCI group. Within each of the groups, cognitive decline is most prominent in the triple-positive group, being already significantly different from the reference group A−/T−/N− from month 18 onwards for the MCI group and from month 12 onwards for the dementia group. In both groups, decline at the final 36 months timepoint was more than 2.5 MMSE points for A+/T+/N+ compared to A+/T−/N−.

Presence of each individual biomarker is significantly associated with cognitive decline, with the CSF Aβ_42_/t-tau ratio having the strongest effect compared to the individual biomarkers.

In the evolution of AD, amyloid-pathology is hypothesized to precede tau-pathology, followed by progressive neurodegeneration [6, 7]. Following this evolution, one would expect Aβ_42_ to be decreased first (A+/T−/N−), followed by an increased p_181_-tau (A+/T+/N−) and finally an elevation of t-tau (A+/T+/N+). The triple-negative class is the class of patients who are unlikely to have AD as a reason for their cognitive problems. The cut-off values for CSF Aβ_42_ used here (i.e., 798 pg/ml) had a relatively high sensitivity (85%) for detecting amyloid-positivity in healthy individuals [14]. Hence, values above this cut-off for Aβ_42_ more or less exclude AD. However, there are exceptions where CSF Aβ_42_ remains high while the ratio of Aβ_42_/Aβ_40_ is low, sometimes referred to as “high amyloid producers” [16, 17]. The amyloid-only positive class are patients who are probably early in the course of AD since t-tau and p_181_-tau only are hypothesized to become abnormal later in the disease course closer to or after the start of cognitive decline. However, in a clinical population, the specificity of an isolated decrease of Aβ_42_ is probably lower than estimated from the healthy individuals derived from amyloid-PET positivity: clinical syndromes other than AD, such as Lewy Body dementia (LBD), normal pressure hydrocephalus (NPH) or vascular dementia, can also cause an isolated Aβ_42_ decrease [18]. Again, the ratio of Aβ_42_/Aβ_40_ more or less resolves this issue. However, in clinical practice we have been using the ratio of Aβ_42_/Aβ_40_ only since 2018 and given the required 3-year follow-up of the current sample, this was not applicable. About 17% of patients was triple positive, meaning their pathological evolution was already at a further stage. Since the lumbar punctures were performed in patients with incipient yet progressive cognitive complaints, this distribution is not surprising.

In the main analysis, the cut-offs were based on [^18^F]flutemetamol amyloid-PET positivity as gold standard. This is based on the neuropathological validity of [^18^F]flutemetamol PET for the detection of moderate-to-high density of neuritic plaques [19]. Neuritic plaques are composed of Aβ amyloid and also contain tau-positive neurites. A neuropathologically validated in vivo measure of tau aggregation may have been preferable for determining the p_181_-tau cut-off. However, an end-of-life validation study is only available for [^18^F]AV1451 at the moment [20]. This A16 study showed a sensitivity of 92–100% for detecting tau accumulation (Braak stage V-VI) with a specificity of 52–90%. This first-generation tau-PET tracer is no longer in use at our center due to its limitations (mainly off-target binding). For neurodegeneration and total tau, an in vivo gold standard is even more problematic in the absence of end-of-life neuropathological validation data and a strict definition of neurodegeneration. However, we examined the effect of the exact p_181_-tau and t-tau cut-offs used by also classifying cases based on a considerably lower cut-off derived from an independent clinical dataset. In this way, the two classifications covered a range of p_181_-tau and t-tau cut-offs commonly used at different centers. Evidently, which p_181_-tau and t-tau cut-offs to select depends on the trade-off between sensitivity and specificity. Regardless of the exact cut-offs used, the conclusion that in a clinical real-life context, the CSF-based ATN classification bears prognostic value for clinical cognitive decline was confirmed.

Depending on the classification scheme, 8.77 to 11.84% of patients showed aberrant values for Aβ_42_ and t-tau with a normal value for p_181_-tau (Fig. 1), while we had no or very few patients with abnormal values for Aβ_42_ and p_181_-tau with a normal t-tau. These results contradict the statements of Jack et al. [7]. It is important to note that the ATN classification is dependent on the assay and on the exact thresholds used for p_181_-tau and t-tau. In the main analysis, the p_181_-tau and t-tau thresholds were based on amyloid-positivity as gold standard in a normal population. We also examined the effect of classifying cases based on an alternative p_181_-tau and t-tau threshold derived from an independent clinical patient cohort. Thresholds are often variable between centers, which is an inherent weakness of the ATN approach. This variance is often most relevant for p_181_-tau, where differences between normal subjects and patients are often small. This can be an explanation for the apparent contradiction between papers and studies, which may therefore have more to do with the thresholds used and highlights one of the main criticisms against the ATN approach. However, regardless of the exact thresholds used, the conclusions regarding the predictive value of CSF-based ATN classification for future cognitive decline remained valid. Further, even though the original ATN scheme included CSF p_181_-tau in the tau (T) category and t-tau in the neurodegeneration (N) category, it is known that CSF p_181_-tau and t-tau are strongly correlated [21]. This is also reflected in our results, since the dichotomized results for p_181_-tau and t-tau differ in only 13.59% to 16.23% of our population, depending on the cut-offs used.

As expected, positivity for p_181_-tau only was very rare (Fig. 1). Furthermore, only a few patients were A−/T+/N+; these classes would probably represent the pure tauopathies rather than AD. Finally, 10 patients (approximately 5% of the study cohort) were positive for neurodegeneration without aberrant Aβ_42_ or p_181_-tau. These biomarker profiles are suggestive of non-AD neurodegenerative disorders, and the patients were eventually diagnosed with corticobasal syndrome (3 patients), frontotemporal dementia (2 patients), no neurodegenerative disorder (4 patients), or unknown (1 patient). The cognitive deficit in the four patients without a neurodegenerative disorder was attributed to left frontotemporal epilepsy, internal medicine disorders (combination of non-alcoholic steatohepatitis with cirrhosis and primary hyperparathyroidism), medication (topiramate for essential tremor, cognitive improvement after discontinuation of this drug), and a final patient with limited premorbid cognition and essential tremor, who first presented in 1994 with objective cognitive symptoms, but subsequently disappeared from follow-up. Cognitive evaluation was stable in 2002, after which the patient disappeared from follow-up again. At re-presentation in 2016, the patient showed a mild deterioration for which further investigations at that time, which could exclude evolution to AD based on CSF biomarkers and [^18^F]FDG-PET. In 2017, a renewed clinical cognitive evaluation was comparable to prior evaluations. Apart from the last two cases, these are all known causes of isolated t-tau increase.

The A−/T−/N+ class shows a pronounced deterioration of MMSE over the 3-year follow-up period, which is driven by the patients diagnosed with corticobasal syndrome. This is a rapidly progressive neurodegenerative disorder with a survival rate of on average only 4–5 years [22]. Of the three patients with a diagnosis of corticobasal syndrome, two were deceased at the moment of this writing, one of which had an autopsy confirming a diagnosis of frontotemporal lobar degeneration-tau (FTLD-tau), subtype corticobasal degeneration (CBD) [23].

The baseline Aβ_42_/t-tau ratio as well as the individual CSF biomarkers showed a significant correlation with the time course of cognitive decline (Table 3, Fig. 3). This significant association was in addition globally valid within ATN classes. A remarkable discrepancy is the strongly inverse correlation of p_181_-tau in the A−/T−/N+ class, where a higher p_181_-tau level is associated with a better cognitive outcome. This is the opposite of what would be expected in AD. This can be explained by the fact that the lower the p_181_-tau value is in this non-AD population, the more it is remote from AD, the more drastically the cognitive decline. This effect is probably driven by the 3 patients with corticobasal syndrome in the A−/T−/N+ class, who had low p_181_-tau values (respectively 52.70 pg/mL, 56.10 pg/mL and 67.30 pg/mL; threshold 87 pg/mL) and steep cognitive decline.

An important point to emphasize is that these CSF biomarkers seem to have a prognostic value, independent of the final clinical diagnosis. In a recent study by Selvackadunco et al. [24], clinical and post mortem histopathological diagnoses matched in only 115 out of 180 patients (64%). Despite recent advances in diagnostic biomarkers, misdiagnosis or incomplete diagnosis for patients with cognitive decline is still a frequent problem. Predicting a prognosis based on solely the clinical diagnosis can thus be unreliable.

The current study demonstrates that CSF biomarker measurements can have not only a diagnostic added value, but can also have important relevance for determining the prognosis of cognitive evolution. These CSF biomarkers have been shown to correlate with cognitive evolution in multiple trials before, both in cognitive normal individuals [25–27] as in patients with MCI due to AD [28, 29] and in clinically probable AD [30]. Our data are in line with these results.

Soldan et al. [31] recently investigated the cognitive evolution among different ATN classes based solely on CSF biomarkers in cognitively normal individuals. Duration of follow-up was an average of 7 years for 85% of participants. They concluded that only the triple-positive class had a higher risk for cognitive deterioration relative to the reference class of triple-negative individuals, and differentiation of T and N had no prognostic value in individuals without cognitive symptoms. As mentioned before, we did see statistically significant and clinically relevant results in not only the triple-positive class, but also in the other classes. This difference can be explained because patients in our study did have cognitive deficits, meaning they were probably further in the stage of their neurodegenerative disorder (AD or non-AD), causing more important differences in cognitive evolution. Furthermore, Ebenau et al. [32] classified 693 participants with subjective cognitive decline (SCD) from the Amsterdam Dementia Cohort and Subjective Cognitive Impairment Cohort according to the ATN class. Results showed that, compared to A−/T−/N−, patients positive for amyloid had a higher risk of having dementia and showed a steeper decline on cognitive tests after a follow-up period of 3 years, similar to our findings. Finally, a study applying the ATN scheme in a memory clinic population was recently published using retrospective data from the ABIDE project (“A” based on amyloid PET, “T” based on p_181_-tau in CSF, “N” based on medial temporal atrophy on MRI). Altomare et al. [33] showed a faster cognitive decline in the A+/T+/N− and A+/T+/N+ classes compared to A−/T−/N− over an average follow-up period of 16 months. Our findings confirm these results and extend them to a CSF-only based classification and a 36-month follow-up period.

## Limitations

Our study has several limitations. We present the outcome from a retrospective study, where all consecutive results from CSF biomarkers were used. However, not every patient seen in the Neurology Memory Clinic UZ/KU Leuven had a lumbar puncture. This investigation was proposed mostly in patients with an unclear diagnosis, an atypical presentation, age of onset below 65 years, or patients who were suspected of another etiology requiring a lumbar puncture, in which cases also CSF biomarkers for AD were determined. This may have led to a selection bias. Further, we only included patients for whom at least three values of MMSE were available to be able to reliably map an evolution in cognitive function; however, we have no notion on the reasons for drop-outs. In addition, we have missing data for the patients who were included, for which statistical analyses corrected using imputation. These data are shown in the Supplementary Data and show very similar results to the analyses without imputation of missing values. Also, MMSE scores are commonly clinically used for follow-up of cognitive function in patients with AD, but have limited sensitivity for detecting change even in AD and are also less suited/sensitive for other neurodegenerative or non-neurodegenerative cognitive disorders [34]. Next, follow-up with MMSE was divided into time intervals of 6-month periods instead of using exact time points. The 6 months’ binning was based on the 6-month intervals of the clinical follow-up visits. For that reason, while a more exact timing of the longitudinal data might have been more elegant, this would not be expected to affect the results significantly given the 6-month intervals of the clinical follow-up.

The ATN classification can be based on multiple biomarkers, including CSF biomarkers but also imaging techniques. In our study, we only used CSF biomarkers and analyzed them in a single laboratory, consistently using the same immuno-assay to classify the patients. Although results from CSF biomarkers can vary between different laboratories, it seems to be more reproducible and less rater-dependent relative to results from MRI or [^18^F]FDG-PET imaging techniques. An inherent disadvantage from the ATN classification is its binary division into classes, while the values of CSF biomarkers include a continuous value, which can be more informative. The necessity to define a cut-off to classify cases is also problematic for the ATN classification. The biological variable does not have a binary distribution, and furthermore, given the inter-laboratory variability of CSF AD biomarker assays and the absence of a neuropathological gold standard, cut-offs may vary between laboratories which impacts on the ATN classification. It is however worth noting that our conclusions remained when we varied the exact p_181_-tau and t-tau cut-offs. Finally, due to the low sample size of some ATN classes, a comprehensive evaluation of the 8 ATN classes was not feasible.

## Conclusion

Based on this retrospective analysis, ATN classification based on CSF biomarkers seems to have a statistical but also clinically relevant prognostic value for the course of cognitive decline over a 3-year period. Relative to the A−/T−/N− class, MMSE declines strongly in patients with the A+ biomarker but surprisingly also in the neurodegeneration-only (A−/T−/N+) group. All individual CSF biomarkers and the CSF Aβ_42_/t-tau ratio showed a significant correlation with cognitive decline. The highest correlation was seen for the CSF Aβ_42_/t-tau ratio. Further confirmation of these results in prospective analyses is needed.

## Supplementary Information

**Additional file 1: Supplementary Table 1.** Difference in MMSE at different time points relative to the reference class (A-/T-/N-) and to the baseline MMSE using linear mixed-effects model with random slope and imputation of missing values. The following CSF thresholds were used: 798 pg/mL for Aβ_42_, 87 pg/mL for p_181_-tau, 465 pg/mL for t-tau. **Supplementary Table 2.** Difference in MMSE at different time points relative to the reference class (A-/T-/N-) and to the baseline MMSE using linear mixed-effects model with random slope. The following CSF thresholds were used: 798 pg/mL for Aβ_42_, 58.9 pg/mL for p_181_-tau, 354 pg/mL for t-tau. **Supplementary Table 3.** Difference in MMSE at different time points relative to the reference class (A-/T-/N-) and to the baseline MMSE using linear mixed-effects model with random slope and imputation of missing values. The following CSF thresholds were used: 798 pg/mL for Aβ_42_, 58.9 pg/mL for p_181_-tau, 354 pg/mL for t-tau. **Supplementary Table 4.** Interaction of individual CSF biomarkers on evolution of MMSE using linear mixed-effects model and imputation of missing values. **Supplementary Table 5.** Distribution of syndrome diagnoses at the moment of the lumbar puncture per ATN class, based on the following cut-off values: 798 pg/mL for Aβ_42_, 87 pg/mL for p_181_-tau, 465 pg/mL for t-tau. MCI = mild cognitive impairment. **Supplementary Table 6.** Difference in MMSE at different time points relative to the reference class (A-/T-/N-) and to the baseline MMSE using linear mixed-effects model with random slope for patients with a syndrome diagnosis of mild cognitive impairment. The following CSF thresholds were used: 798 pg/mL for Aβ_42_, 87 pg/mL for p_181_-tau, 465 pg/mL for t-tau. **Supplementary Table 7.** Difference in MMSE at different time points relative to the reference class (A-/T-/N-) and to the baseline MMSE using linear mixed-effects model with random slope for patients with a syndrome diagnosis of dementia. The following CSF thresholds were used: 798 pg/mL for Aβ_42_, 87 pg/mL for p_181_-tau, 465 pg/mL for t-tau. **Supplementary Table 8.** Distribution of final etiological diagnoses per ATN class based on the clinical-diagnostic investigation including cerebrospinal fluid biomarkers. AD = Alzheimer’s disease. Other neurodeg = non-AD neurodegenerative disorders, including FTD (frontotemporal dementia), LBD (Lewy Body disease) and CBS (corticobasal syndrome). Non-neurodeg = no neurodegenerative disorders. ‘No diagnosis’ is reserved for patients without a clear diagnosis after standard clinical work-up.

## Data Availability

The datasets during and/or analyzed during the current study available from the corresponding author on reasonable request.
