# Supplementary material for: Prognostic value of amyloid/tau/neurodegeneration (ATN) classification based on diagnostic cerebrospinal fluid samples for Alzheimer’s disease
Source: Alzheimers Res Ther. 2021 Apr 20;13:84. doi: 10.1186/s13195-021-00817-4 (PMC8059197; doi:10.1186/s13195-021-00817-4)
Supplement: Supplementary file 1 — Additional file 1: Supplementary Table 1. Difference in MMSE at different time points relative to the reference class (A-/T-/N-) and to the baseline MMSE using linear mixed-effects model with random slope and imputation of missing values. The following CSF thresholds were used: 798 pg/mL for Aβ42, 87 pg/mL for p181-tau, 465 pg/mL for t-tau. Supplementary Table 2. Difference in MMSE at different time points relative to the reference class (A-/T-/N-) and to the baseline MMSE using linear mixed-effects model with random slope. The following CSF thresholds were used: 798 pg/mL for Aβ42, 58.9 pg/mL for p181-tau, 354 pg/mL for t-tau. Supplementary Table 3. Difference in MMSE at different time points relative to the reference class (A-/T-/N-) and to the baseline MMSE using linear mixed-effects model with random slope and imputation of missing values. The following CSF thresholds were used: 798 pg/mL for Aβ42, 58.9 pg/mL for p181-tau, 354 pg/mL for t-tau. Supplementary Table 4. Interaction of individual CSF biomarkers on evolution of MMSE using linear mixed-effects model and imputation of missing values. Supplementary Table 5. Distribution of syndrome diagnoses at the moment of the lumbar puncture per ATN class, based on the following cut-off values: 798 pg/mL for Aβ42, 87 pg/mL for p181-tau, 465 pg/mL for t-tau. MCI = mild cognitive impairment. Supplementary Table 6. Difference in MMSE at different time points relative to the reference class (A-/T-/N-) and to the baseline MMSE using linear mixed-effects model with random slope for patients with a syndrome diagnosis of mild cognitive impairment. The following CSF thresholds were used: 798 pg/mL for Aβ42, 87 pg/mL for p181-tau, 465 pg/mL for t-tau. Supplementary Table 7. Difference in MMSE at different time points relative to the reference class (A-/T-/N-) and to the baseline MMSE using linear mixed-effects model with random slope for patients with a syndrome diagnosis of dementia. The following CSF thresholds were used: 798 [file 13195_2021_817_MOESM1_ESM.docx]

**Supplementary data**

| **ATN Class (n)** | **Time point** | **Difference in MMSE relative to baseline value** | **95% CI** | ***t*** | ***P*** |
| --- | --- | --- | --- | --- | --- |
| A-/T-/N- (n=73) | Time 6 | 0.23 | -0.73, 1.20 | 0.47 | 0.6400 |
|  | Time 12 | 0.07 | -0.90, 1.03 | 0.14 | 0.8906 |
|  | Time 18 | 0.25 | -0.72, 1.21 | 0.50 | 0.6204 |
|  | Time 24 | -0.93 | -1.90, 0.03 | -1.87 | 0.0615 |
|  | Time 30 | -1.77 | -2.73, -0.80 | -3.55 | **0.0004** |
|  | Time 36 | -1.38 | -2.35, -0.42 | -2.78 | **0.0055** |
| **ATN Class (n)** | **Time point** | **Difference in MMSE relative to value predicted based on A-/T-/N- at that time point and baseline MMSE** | **95% CI** | ***t*** | ***P*** |
| A+/T-/N- (n=76) | Time 6 | -0.69 | -2.04, 0.66 | -0.99 | 0.3200 |
|  | Time 12 | -1.07 | -2.42, 0.28 | -1.53 | 0.1255 |
|  | Time 18 | -1.79 | -3.14, -0.43 | -2.56 | **0.0105** |
|  | Time 24 | -1.73 | -3.08, -0.38 | -2.48 | **0.0134** |
|  | Time 30 | -1.42 | -2.77, -0.66 | -2.03 | **0.0422** |
|  | Time 36 | -1.41 | -2.76, -0.05 | -2.02 | **0.0439** |
| A+/T+/N+ (n=39) | Time 6 | -0.82 | -2.46, 0.81 | -0.98 | 0.3297 |
|  | Time 12 | -2.27 | -3.91, -0.64 | -2.70 | **0.0071** |
|  | Time 18 | -3.96 | -5.60, -2.33 | -4.70 | **0.0000** |
|  | Time 24 | -4.38 | -6.01, -2.74 | -5.19 | **0.0000** |
|  | Time 30 | -2.59 | -4.23, -0.96 | -3.07 | **0.0022** |
|  | Time 36 | -4.00 | -5.64, -2.37 | -4.74 | **0.0000** |
| A+/T-/N+ (n=27) | Time 6 | 0.06 | -1.79, 1.92 | 0.07 | 0.9472 |
|  | Time 12 | -0.33 | -2.18, 1.53 | -0.34 | 0.7323 |
|  | Time 18 | -2.25 | -4.10, -0.39 | -2.35 | **0.0192** |
|  | Time 24 | -1.99 | -3.85, -0.14 | -2.08 | **0.0375** |
|  | Time 30 | -1.16 | -3.02, 0.70 | -1.21 | 0.2266 |
|  | Time 36 | -2.62 | -4.47, -0.76 | -2.73 | **0.0064** |
| A-/T-/N+ (n=10) | Time 6 | 0.17 | -2.61, 2.95 | 0.12 | 0.9072 |
|  | Time 12 | -1.57 | -4.35, 1.21 | -1.09 | 0.2743 |
|  | Time 18 | -3.35 | -6.13, -0.57 | -2.33 | **0.0198** |
|  | Time 24 | -3.77 | -6.55, -0.99 | -2.63 | **0.0087** |
|  | Time 30 | -3.53 | -6.31, -0.75 | -2.46 | **0.0139** |
|  | Time 36 | -5.02 | -7.80, -2.24 | -3.50 | **0.0005** |

**Supplementary Table 1.** Difference in MMSE at different time points relative to the reference class (A-/T-/N-) and to the baseline MMSE using linear mixed-effects model with random slope and imputation of missing values. The following CSF thresholds were used: 798 pg/mL for Aβ_42_, 87 pg/mL for p_181_-tau, 465 pg/mL for t-tau.

MMSE: Mini Mental State Examination. CI: confidence interval. The model is corrected for age and sex. Time 6 corresponds to the period 0-0.5 year; time 12 corresponds to the period 0.5-1 year; time 18 corresponds to the period 1-1.5 year; time 24 corresponds to the period 1.5-2 year; time 30 corresponds to the period 2-2.5 year; time 36 corresponds to the period 2.5-3 year. Significant *p*-values are represented in bold.

| **ATN Class (n)** | **Time point** | **Difference in MMSE relative to baseline value** | **95% CI** | ***t*** | ***P*** |
| --- | --- | --- | --- | --- | --- |
| A-/T-/N- (n=63) | Time 6 | 0.63 | -0.57, 1.83 | 1.01 | 0.3140 |
|  | Time 12 | 0.26 | -0.89, 1.40 | 0.43 | 0.6651 |
|  | Time 18 | -0.06 | -1.30, 1.18 | -0.09 | 0.9279 |
|  | Time 24 | -0.73 | -1.91, 0.46 | -1.18 | 0.2382 |
|  | Time 30 | -1.43 | -2.80, -0.06 | -2.02 | **0.0442** |
|  | Time 36 | -0.99 | -2.36, 0.38 | -1.40 | 0.1623 |
| **ATN Class (n)** | **Time point** | **Difference in MMSE relative to value predicted based on A-/T-/N- at that time point and baseline MMSE** | **95% CI** | ***t*** | ***P*** |
| A+/T-/N- (n=57) | Time 6 | -0.97 | -2.73, 0.80 | -1.06 | 0.2909 |
|  | Time 12 | -1.10 | -2.76, 0.56 | -1.27 | 0.2039 |
|  | Time 18 | -1.09 | -2.80, 0.61 | -1.23 | 0.2179 |
|  | Time 24 | -2.03 | -3.76, -0.30 | -2.27 | **0.0238** |
|  | Time 30 | -1.88 | -3.75, -0.02 | -1.94 | 0.0522 |
|  | Time 36 | -1.86 | -3.78, 0.06 | -1.87 | 0.0620 |
| A+/T+/N+ (n=63) | Time 6 | -1.16 | -2.84, 0.53 | -1.32 | 0.1856 |
|  | Time 12 | -1.86 | -3.43, -0.30 | -2.30 | **0.0218** |
|  | Time 18 | -2.94 | -4.58, -1.30 | -3.46 | **0.0006** |
|  | Time 24 | -3.20 | -4.84, -1.55 | -3.73 | **0.0002** |
|  | Time 30 | -3.71 | -5.51, -1.90 | -3.95 | **0.0001** |
|  | Time 36 | -4.70 | -6.56, -2.83 | -4.86 | **0.0000** |
| A+/T-/N+ (n=20) | Time 6 | -0.08 | -2.63, 2.48 | -0.06 | 0.9541 |
|  | Time 12 | -1.04 | -3.17, 1.10 | -0.93 | 0.3503 |
|  | Time 18 | -3.20 | -5.47, -0.93 | -2.71 | **0.0068** |
|  | Time 24 | -2.91 | -5.24, -0.59 | -2.41 | **0.0161** |
|  | Time 30 | -3.20 | -5.77, -0.63 | -2.39 | **0.0169** |
|  | Time 36 | -2.25 | -4.89, 0.39 | -1.64 | 0.1007 |
| A-/T+/N+ (n=14) | Time 6 | -0.41 | -3.15, 2.34 | -0.28 | 0.7760 |
|  | Time 12 | -0.56 | -3.28, 2.16 | -0.40 | 0.6899 |
|  | Time 18 | -0.44 | -3.30, 2.42 | -0.30 | 0.7659 |
|  | Time 24 | -2.25 | -5.58, 1.08 | -1.30 | 0.1938 |
|  | Time 30 | -0.21 | -6.77, 6.35 | -0.06 | 0.9502 |
|  | Time 36 | -4.36 | -9.18, 0.47 | -1.74 | 0.0826 |

**Supplementary Table 2.** Difference in MMSE at different time points relative to the reference class (A-/T-/N-) and to the baseline MMSE using linear mixed-effects model with random slope. The following CSF thresholds were used: 798 pg/mL for Aβ_42_, 58.9 pg/mL for p_181_-tau, 354 pg/mL for t-tau.

MMSE: Mini Mental State Examination. CI: confidence interval. The model is corrected for age and sex. Time 6 corresponds to the period 0-0.5 year; time 12 corresponds to the period 0.5-1 year; time 18 corresponds to the period 1-1.5 year; time 24 corresponds to the period 1.5-2 year; time 30 corresponds to the period 2-2.5 year; time 36 corresponds to the period 2.5-3 year. Significant *p*-values are represented in bold.

| **ATN Class (n)** | **Time point** | **Difference in MMSE relative to baseline value** | **95% CI** | ***t*** | ***P*** |
| --- | --- | --- | --- | --- | --- |
| A-/T-/N- (n=63) | Time 6 | 0.43 | -0.63, 1.49 | 0.78 | 0.4347 |
|  | Time 12 | 0.56 | -0.51, 1.62 | 1.01 | 0.3113 |
|  | Time 18 | 0.49 | -0.57, 1.55 | 0.90 | 0.3698 |
|  | Time 24 | -0.48 | -1.54, 0.59 | -0.87 | 0.3855 |
|  | Time 30 | -0.54 | -1.60, 0.52 | -0.98 | 0.3253 |
|  | Time 36 | -0.70 | -1.76, 0.36 | -1.27 | 0.2031 |
| **ATN Class (n)** | **Time point** | **Difference in MMSE relative to value predicted based on A-/T-/N- at that time point and baseline MMSE** | **95% CI** | ***t*** | ***P*** |
| A+/T-/N- (n=57) | Time 6 | -0.88 | -2.43, 0.66 | -1.11 | 0.2665 |
|  | Time 12 | -1.22 | -2.76, 0.32 | -1.54 | 0.1248 |
|  | Time 18 | -1.69 | -3.23, -0.14 | -2.12 | **0.0344** |
|  | Time 24 | -1.86 | -3.40, -0.31 | -2.33 | **0.0198** |
|  | Time 30 | -2.46 | -4.00, -0.92 | -3.09 | **0.0020** |
|  | Time 36 | -2.49 | -4.04, -0.95 | -3.13 | **0.0018** |
| A+/T+/N+ (n=63) | Time 6 | -1.52 | -3.03, -0.02 | -1.96 | **0.0497** |
|  | Time 12 | -2.19 | -3.69, -0.69 | -2.82 | **0.0048** |
|  | Time 18 | -3.52 | -5.03, -2.02 | -4.54 | **0.0000** |
|  | Time 24 | -3.60 | -5.11, -2.10 | -4.65 | **0.0000** |
|  | Time 30 | -4.63 | -6.14, -3.13 | -5.98 | **0.0000** |
|  | Time 36 | -5.21 | -6.71, -3.70 | -6.71 | **0.0000** |
| A+/T-/N+ (n=20) | Time 6 | 0.02 | -2.14, 2.19 | 0.02 | 0.9847 |
|  | Time 12 | -1.46 | -3.62, 0.71 | -1.30 | 0.1929 |
|  | Time 18 | -3.49 | -5.66, -1.33 | -3.13 | **0.0018** |
|  | Time 24 | -3.42 | -5.59, -1.26 | -3.06 | **0.0022** |
|  | Time 30 | -4.11 | -6.28, -1.95 | -3.68 | **0.0002** |
|  | Time 36 | -4.60 | -6.77, -2.44 | -4.12 | **0.0000** |
| A-/T+/N+ (n=14) | Time 6 | 0.07 | -2.42, 2.56 | 0.06 | 0.9557 |
|  | Time 12 | -0.41 | -2.91, 2.08 | -0.32 | 0.7484 |
|  | Time 18 | -0.99 | -3.48, 1.50 | -0.77 | 0.4407 |
|  | Time 24 | -1.81 | -4.30, 0.68 | -1.41 | 0.1597 |
|  | Time 30 | -2.75 | -5.24, -0.25 | -2.13 | **0.0330** |
|  | Time 36 | -1.94 | -4.44, 0.55 | -1.51 | 0.1309 |

**Supplementary Table 3.** Difference in MMSE at different time points relative to the reference class (A-/T-/N-) and to the baseline MMSE using linear mixed-effects model with random slope and imputation of missing values. The following CSF thresholds were used: 798 pg/mL for Aβ_42_, 58.9 pg/mL for p_181_-tau, 354 pg/mL for t-tau.

MMSE: Mini Mental State Examination. CI: confidence interval. The model is corrected for age and sex. Time 6 corresponds to the period 0-0.5 year; time 12 corresponds to the period 0.5-1 year; time 18 corresponds to the period 1-1.5 year; time 24 corresponds to the period 1.5-2 year; time 30 corresponds to the period 2-2.5 year; time 36 corresponds to the period 2.5-3 year. Significant *p*-values are represented in bold.

| **CSF biomarker** | **Time point** | **Interaction** | **95% CI** | ***t*** | ***P*** |
| --- | --- | --- | --- | --- | --- |
| Aβ_42_/t-tau | Time 6 | 0.1179 | -0.1580, 0.3939 | 0.8341 | 0.4044 |
|  | Time 12 | 0.2838 | 0.0079, 0.5598 | 2.0074 | **0.0449** |
|  | Time 18 | 0.7480 | 0.4721, 1.0240 | 5.2906 | **0.0000** |
|  | Time 24 | 0.7412 | 0.4652, 1.0171 | 5.2421 | **0.0000** |
|  | Time 30 | 0.5450 | 0.2690, 0.8209 | 3.8542 | **0.0001** |
|  | Time 36 | 0.8122 | 0.5362, 1.0881 | 5.7441 | **0.0000** |
| Aβ_42_ | Time 6 | 0.0009 | -0.0006, 0.0025 | 1.2167 | 0.2239 |
|  | Time 12 | 0.0014 | -0.0002, 0.0029 | 1.7494 | 0.0804 |
|  | Time 18 | 0.0029 | 0.0014, 0.0045 | 3.7673 | **0.0002** |
|  | Time 24 | 0.0026 | 0.0011, 0.0041 | 3.3747 | **0.0008** |
|  | Time 30 | 0.0015 | -0.0000, 0.0030 | 1.9270 | 0.0542 |
|  | Time 36 | 0.0025 | 0.0010, 0.0040 | 3.1890 | **0.0015** |
| p_181_-tau | Time 6 | -0.0025 | -0.0177, 0.0128 | -0.3150 | 0.7528 |
|  | Time 12 | -0.0140 | -0.0292, 0.0013 | -1.7827 | 0.0749 |
|  | Time 18 | -0.0252 | -0.0405, -0.0100 | -3.2235 | **0.0013** |
|  | Time 24 | -0.0282 | -0.0435, -0.0129 | -3.6013 | **0.0003** |
|  | Time 30 | -0.0162 | -0.0312, -0.0009 | -2.0730 | **0.0384** |
|  | Time 36 | -0.0309 | -0.0462, -0.0157 | -3.9530 | **0.0001** |
| t-tau | Time 6 | -0.0003 | -0.0021, 0.0015 | -0.2922 | 0.7702 |
|  | Time 12 | -0.0017 | -0.0035, 0.0001 | -1.8687 | 0.0619 |
|  | Time 18 | -0.0041 | -0.0059, -0.0023 | -4.4017 | **0.0000** |
|  | Time 24 | -0.0047 | -0.0066, -0.0029 | -5.1002 | **0.0000** |
|  | Time 30 | -0.0025 | -0.0043, -0.0007 | -2.7061 | **0.0069** |
|  | Time 36 | -0.0045 | -0.0063, -0.0027 | -4.8416 | **0.0000** |

**Supplementary Table 4.** Interaction of individual CSF biomarkers on evolution of MMSE using linear mixed-effects model and imputation of missing values.

CSF: cerebrospinal fluid. CI: confidence interval. Model is corrected for age and sex. Time 6 corresponds to the period 0-0.5 year; time 12 corresponds to the period 0.5-1 year; time 18 corresponds to the period 1-1.5 year; time 24 corresponds to the period 1.5-2year; time 30 corresponds to the period 2-2.5 year; time 36 corresponds to the period 2.5-3 year. Significant *p*-values are represented in bold.

| **ATN class** | **Syndrome diagnosis per class** | | |
| --- | --- | --- | --- |
|  | N_total_ | N_MCI_ (% of total MCI) | N_dementia_ (% of total dementia) |
| A-/T-/N- | 73 | 27 (34.18) | 46 (30.87) |
| A+/T-/N- | 76 | 27 (34.18 | 49 (32.89) |
| A+/T+/N+ | 39 | 10 (12.66) | 29 (19.46) |
| A+/T-/N+ | 27 | 10 (12.66) | 17 (11.41) |
| A-/T-/N+ | 10 | 4 (5.06) | 6 (4.03) |
| A-/T+/N+ | 3 | 1 (1.27) | 2 (1.34) |
| A-/T+/N- |  |  |  |
| A+/T+/N- |  |  |  |
| Total | 228 | 79 | 149 |

**Supplementary Table 5.** Distribution of syndrome diagnoses at the moment of the lumbar puncture per ATN class, based on the following cut-off values: 798 pg/mL for Aβ_42_, 87 pg/mL for p_181_-tau, 465 pg/mL for t-tau. MCI = mild cognitive impairment.

| **ATN Class (n)** | **Time point** | **Difference in MMSE relative to baseline value** | **95% CI** | ***t*** | ***P*** |
| --- | --- | --- | --- | --- | --- |
| A-/T-/N- (n=27) | Time 6 | -0.70 | -1.96, 0.56 | -1.06 | 0.2877 |
|  | Time 12 | -0.30 | -1.56, 0.96 | -0.45 | 0.6542 |
|  | Time 18 | 0.00 | -1.26, 1.26 | 0.00 | 1.0000 |
|  | Time 24 | -0.89 | -2.15, 0.37 | -1.34 | 0.1794 |
|  | Time 30 | -1.22 | -2.48, 0.04 | -1.85 | 0.0652 |
|  | Time 36 | -1.00 | -2.26, 0.26 | -1.51 | 0.1311 |
| **ATN Class (n)** | **Time point** | **Difference in MMSE relative to value predicted based on A-/T-/N- at that time point and baseline MMSE** | **95% CI** | ***t*** | ***P*** |
| A+/T-/N- (n=27) | Time 6 | -0.63 | -2.41, 1.15 | -0.67 | 0.5010 |
|  | Time 12 | -1.22 | -3.01, 0.56 | -1.31 | 0.1918 |
|  | Time 18 | -2.19 | -3.97, -0.40 | -2.34 | **0.0199** |
|  | Time 24 | -2.81 | -4.60, -1.03 | -3.01 | **0.0028** |
|  | Time 30 | -2.07 | -3.86, -0.29 | -2.22 | **0.0270** |
|  | Time 36 | -1.78 | -3.56, 0.01 | -1.90 | 0.0579 |
| A+/T+/N+ (n=10) | Time 6 | -0.40 | -2.82, 2.03 | -0.31 | 0.7554 |
|  | Time 12 | -1.60 | -4.03, 0.82 | -1.26 | 0.2079 |
|  | Time 18 | -4.20 | -6.63, -1.77 | -3.30 | **0.0010** |
|  | Time 24 | -4.21 | -6.64, -1.79 | -3.31 | **0.0010** |
|  | Time 30 | -4.08 | -6.50, -1.65 | -3.21 | **0.0014** |
|  | Time 36 | -4.90 | -7.33, -2.47 | -3.85 | **0.0001** |
| A+/T-/N+ (n=10) | Time 6 | 0.80 | -1.62, 3.23 | 0.63 | 0.5277 |
|  | Time 12 | 0.20 | -2.23, 2.62 | 0.15 | 0.8774 |
|  | Time 18 | -0.60 | -3.03, 1.83 | -0.47 | 0.6372 |
|  | Time 24 | -2.01 | -4.44, 0.41 | -1.58 | 0.1145 |
|  | Time 30 | -1.88 | -4.30, 0.55 | -1.48 | 0.1405 |
|  | Time 36 | -3.30 | -5.73, -0.87 | -2.60 | **0.0098** |

**Supplementary Table 6.** Difference in MMSE at different time points relative to the reference class (A-/T-/N-) and to the baseline MMSE using linear mixed-effects model with random slope for patients with a syndrome diagnosis of mild cognitive impairment. The following CSF thresholds were used: 798 pg/mL for Aβ_42_, 87 pg/mL for p_181_-tau, 465 pg/mL for t-tau.

MMSE: Mini Mental State Examination. CI: confidence interval.

| **ATN Class (n)** | **Time point** | **Difference in MMSE relative to baseline value** | **95% CI** | ***t*** | ***P*** |
| --- | --- | --- | --- | --- | --- |
| A-/T-/N- (n=46) | Time 6 | 0.78 | -0.51, 2.08 | 1.17 | 0.2437 |
|  | Time 12 | 0.28 | -1.01, 1.58 | 0.42 | 0.6737 |
|  | Time 18 | 0.39 | -0.91, 1.69 | 0.58 | 0.5598 |
|  | Time 24 | -0.96 | -2.25, 0.34 | -1.43 | 0.1543 |
|  | Time 30 | -2.09 | -3.38, -0.79 | -3.11 | **0.0019** |
|  | Time 36 | -1.61 | -2.91, -0.31 | -2.40 | **0.0167** |
| **ATN Class (n)** | **Time point** | **Difference in MMSE relative to value predicted based on A-/T-/N- at that time point and baseline MMSE** | **95% CI** | ***t*** | ***P*** |
| A+/T-/N- (n=49) | Time 6 | -0.76 | -2.57, 1.04 | -0.82 | 0.4147 |
|  | Time 12 | -1.00 | -2.80, 0.81 | -1.07 | 0.2862 |
|  | Time 18 | -1.57 | -3.38, 0.23 | -1.69 | 0.0921 |
|  | Time 24 | -1.13 | -2.93, 0.68 | -1.20 | 0.2287 |
|  | Time 30 | -1.04 | -2.84, 0.77 | -1.11 | 0.2679 |
|  | Time 36 | -1.19 | -2.99, 0.62 | -1.27 | 0.2041 |
| A+/T+/N+ (n=29) | Time 6 | -1.20 | -3.28, 0.89 | -1.11 | 0.2678 |
|  | Time 12 | -2.59 | -4.68, -0.51 | -2.40 | **0.0165** |
|  | Time 18 | -3.94 | -6.03, -1.86 | -3.65 | **0.0003** |
|  | Time 24 | -4.42 | -6.51, -2.34 | -4.10 | **0.0000** |
|  | Time 30 | -1.95 | -4.03, 0.14 | -1.81 | 0.0714 |
|  | Time 36 | -3.60 | -5.68, -1.51 | -3.34 | **0.0009** |
| A+/T-/N+ (n=17) | Time 6 | -0.37 | -2.87, 2.13 | -0.29 | 0.7741 |
|  | Time 12 | -0.64 | -3.13, 1.86 | -0.49 | 0.6227 |
|  | Time 18 | -3.21 | -5.71, -0.72 | -2.49 | **0.0130** |
|  | Time 24 | -1.98 | -4.48, 0.51 | -1.54 | 0.1247 |
|  | Time 30 | -0.74 | -3.23, 1.76 | -0.57 | 0.5686 |
|  | Time 36 | -2.21 | -4.71, 0.28 | -1.72 | 0.0867 |

**Supplementary Table 7.** Difference in MMSE at different time points relative to the reference class (A-/T-/N-) and to the baseline MMSE using linear mixed-effects model with random slope for patients with a syndrome diagnosis of dementia. The following CSF thresholds were used: 798 pg/mL for Aβ_42_, 87 pg/mL for p_181_-tau, 465 pg/mL for t-tau.

MMSE: Mini Mental State Examination. CI: confidence interval.

| **ATN class** | **Etiological diagnosis per class according to high tau-thresholds*** | | | | |
| --- | --- | --- | --- | --- | --- |
|  | N_total_ | N_AD_ (%) | N_other neurodeg_ (%) | N_non-neurodeg_ (%) | N_no diagnosis_ (%) |
| A-/T-/N- | 73 | 0 (0) | 16 (21.92) | 42 (57.53) | 15 (20.55) |
| A+/T-/N- | 76 | 44 (57.89) | 12 (15.79) | 17 (22.37) | 3 (3.95) |
| A+/T+/N+ | 39 | 38 (97.44) | 0 (0) | 0 (0) | 1 (2.56) |
| A+/T-/N+ | 27 | 23 (85.19) | 4 (14.81) | 0 (0) | 0 (0) |
| A-/T-/N+ | 10 | 0 (0) | 5 (50) | 4 (40) | 1 (10) |
| A-/T+/N+ | 3 | 0 (0) | 1 (33.33) | 2 (66.67) | 0 (0) |
| A-/T+/N- |  |  |  |  |  |
| A+/T+/N- |  |  |  |  |  |
| Total | 228 | 105 (46.05) | 38 (16.67) | 65 (28.51) | 20 (8.77) |
| **ATN class** | **Etiological diagnosis per class according to lower tau-thresholds**** | | | | |
|  | N_total_ | N_AD_ (%) | N_other neurodeg_ (%) | N_non-neurodeg_ (%) | N_no diagnosis_ (%) |
| A-/T-/N- | 63 | 0 (0) | 12 (19.05) | 38 (60.32) | 13 (20.63) |
| A+/T-/N- | 57 | 27 (47.37) | 11 (19.30) | 17 (29.82) | 2 (3.51) |
| A+/T+/N+ | 63 | 61 (96.83) | 1 (1.59) | 0 (0) | 1 (1.59) |
| A+/T-/N+ | 20 | 16 (80) | 4 (20) | 0 (0) | 0 (0) |
| A-/T-/N+ | 5 | 0 (0) | 5 (100) | 0 (0) | 0 (0) |
| A-/T+/N+ | 14 | 0 (0) | 4 (28.57) | 7 (50) | 3 (21.43) |
| A-/T+/N- | 4 | 0 (0) | 1 (25) | 3 (75) | 0 (0) |
| A+/T+/N- | 2 | 1 (50) | 0 (0) | 0 (0) | 1 (50) |
| Total | 228 | 105 (46.05) | 38 (16.67) | 65 (28.51) | 20 (8.77) |

**Supplementary Table 8.** Distribution of final etiological diagnoses per ATN class based on the clinical-diagnostic investigation including cerebrospinal fluid biomarkers. AD = Alzheimer’s disease. Other neurodeg = non-AD neurodegenerative disorders, including FTD (frontotemporal dementia), LBD (Lewy Body disease) and CBS (corticobasal syndrome). Non-neurodeg = no neurodegenerative disorders. ‘No diagnosis’ is reserved for patients without a clear diagnosis after standard clinical work-up.

* The cut-off values used to define the ATN classes were 798 pg/mL for Aβ_42_, 87 pg/mL for p_181_-tau, 465 pg/mL for t-tau.

** The cut-off value is the same for Aβ_42_, but lowered for tau: 58.9 pg/mL for p_181_-tau, 354 pg/mL for t-tau.
